# A Fast Grasp Planning Algorithm for Humanoid Robot Hands

**DOI:** 10.3390/biomimetics9100599

**Published:** 2024-10-04

**Authors:** Ziqi Liu, Li Jiang, Ming Cheng

**Affiliations:** State Key Laboratory of Robotics and System, School of Mechatronics Engineering, Harbin Institute of Technology, Harbin 150008, China; liuzqcn@163.com (Z.L.); mingcheng@hit.edu.cn (M.C.)

**Keywords:** grasp planning, force-closure grasps, multi-fingered robot hand

## Abstract

Grasp planning is crucial for robots to perform precision grasping tasks, where determining the grasp points significantly impacts the performance of the robotic hand. Currently, the majority of grasp planning methods based on analytic approaches solve the problem by transforming it into a nonlinear constrained planning problem. This method often requires performing convex hull computations, which tend to have high computational complexity. This paper proposes a new algorithm for calculating multi-finger force-closure grasps of three-dimensional objects based on humanoid multi-fingered hands. Firstly, sufficient conditions for the multi-finger force-closure grasps of three-dimensional objects are derived from a point contact model with friction. These three-dimensional force-closure conditions are then transformed into two-dimensional plane conditions, leading to a simple algorithm for multi-finger force-closure determination. This method is purely based on geometric analysis, resulting in low computational demands and enabling the rapid assessment of force-closure grasps, which are beneficial for real-time applications. Finally, the algorithm is validated through two case studies, demonstrating its feasibility and effectiveness.

## 1. Introduction

Humanoid robots have entered a phase of vigorous development in recent years. With advancements in technology, robotic hands have become increasingly complex and more similar to human hands, such as the Shadow hand [1], the Schunk hand [2], the RoBioSS hand [3], and the HIT/DLR II robot hand [4]. These dexterous hands typically feature four–five fingers and numerous degrees of freedom, and this high level of flexibility enables them to be used in a wide range of task scenarios [5]. Among these, grasping capability is the most fundamental, influential, and challenging function of multi-fingered dexterous hands [6,7,8]. The process of grasping objects with a dexterous hand is achieved through two levels: the planning layer and the control layer. A crucial part of the planning layer is the planning of grasping positions. Analytic approaches and empirical grasp planning methods are considered in grasp synthesis problems [9]. The former is model-based and is oftentimes limited to specific application scopes. The latter is data-driven and depends heavily on demonstrations, and thus suffers from generalization issues [10].

For data-driven methods, a large amount of data must be used to train grasping strategies through techniques such as deep learning [11,12,13,14]. This approach addresses modeling difficulties by imitating grasping gestures and is suitable for grasping new objects. Therefore, data-intensity is fundamental to implementation, requiring extensive data collection to train grasping strategies, which is labor-intensive [15]. Modern methods usually focus on this aspect. However, although these methods can reproduce and demonstrate different grasping techniques, they are difficult to generalize to arbitrary hand kinematics and often do not consider the resulting physical stability.

In grasping tasks, our goal is to counterbalance any external wrenches with our grasp, thereby achieving force closure. Determining force closure is a critical issue in grasp planning. However, the friction cone constraint is a nonlinear inequality constraint that assesses force closure challenges [6]. Salisbury and Roth [16] first conducted research on force closure, and they proposed that the primitive contact wrench generated by the contact force positively spanning the whole wrench space is a sufficient and necessary condition for force closure.

The critical challenge in the analysis method is the force-closure grasp test. The analytical method generally realizes force closure as a physical constraint, but the force-closure test is expensive. The force-closure problem is usually transformed into a nonlinear constraint programming problem, which needs to be solved iteratively, so many methods are used to simplify the search space. Liu et al. [17] tested force-closed grasping based on linear programming. However, this algorithm can only be applied to three-dimensional frictionless grasping and two-dimensional grasping. Zheng and Qian [18,19] improved this ray method to simplify the testing of force closure, implementing the algorithm in a more efficient manner. Zhu [20] proposed an algorithm based on Q distance to calculate the force-closing grasp of three-dimensional objects with any number of contact points. Zheng [20] introduced a new approach for the optimal grasping of discrete point sets, measuring grasp quality using the object wrench and extending it to real scenarios. Xu et al. [10] formulated a fast and differentiable force-closure estimator, capable of producing diverse and physically stable grasps with arbitrary hand structures, without any training data.

There is also a method that primarily achieves this through physical constraint analysis and geometric calculations. It has been demonstrated that four and seven fingers are sufficient to accomplish two-dimensional and three-dimensional force closure in frictionless grasps, respectively. With point friction contact, three and four fingers are sufficient conditions to attain two-dimensional and three-dimensional force closures, respectively [21]. For specific and unique object shapes (such as spheres), three fingers can allow for force closure in the particular case of the conditions mentioned above. In frictionless and two-dimensional scenarios, the calculation of force closure is relatively straightforward. However, the calculation of force closure is more complex for frictional and three-dimensional multi-fingered grasping.

Nguyen [22] proposed the concept of the independent contact region and directly constructed force-closed grasping, proving that most non-marginal equilibrium grasping belongs to the category of force-closure grasping. Ponce et al. [23,24,25] investigated the necessity of three-finger and four-finger force-closure synthesis for polyhedral objects, transforming the problem into one of projecting polyhedrons onto linear subspaces, and proposed force-closure characteristics for three-finger and four-finger grasps. Based on this, Li et al. [16] proposed a particularly simple and appealing algorithm for calculating attractive three-finger force-closure grasps. This algorithm simplifies the force-closure problem in frictional grasps for two-dimensional and three-dimensional objects into a planar problem for analysis. The algorithm is based on geometric analysis and requires little algebraic computation. It can achieve a fast force closure test in grasping synthesis calculated by a genetic algorithm [15,26]. Bounab et al. [27] proposed a new force-closure grasping proposition for multi-fingered hands to grasp two-dimensional objects based on geometric calculations.

This paper analyzes the contact model for multi-fingered grasping and provides the two-dimensional force-closure conditions and proof for four-finger grasping under antipodal grasp. It presents a force-closure algorithm for four-finger grasping based solely on geometric methods, considering various conditions for achieving a two-dimensional force equilibrium.

## 2. Problem Formulation

### 2.1. Contact Model

The goal of grasp planning problems is to determine suitable contact points, which are essentially contact point layout problems. When a multi-fingered hand contacts an object, the contact models can be divided into three cases: frictionless point contact, frictional point contact, and soft finger contact. The situations considered in this paper are all scenarios of point friction contact.

**Figure 1:**
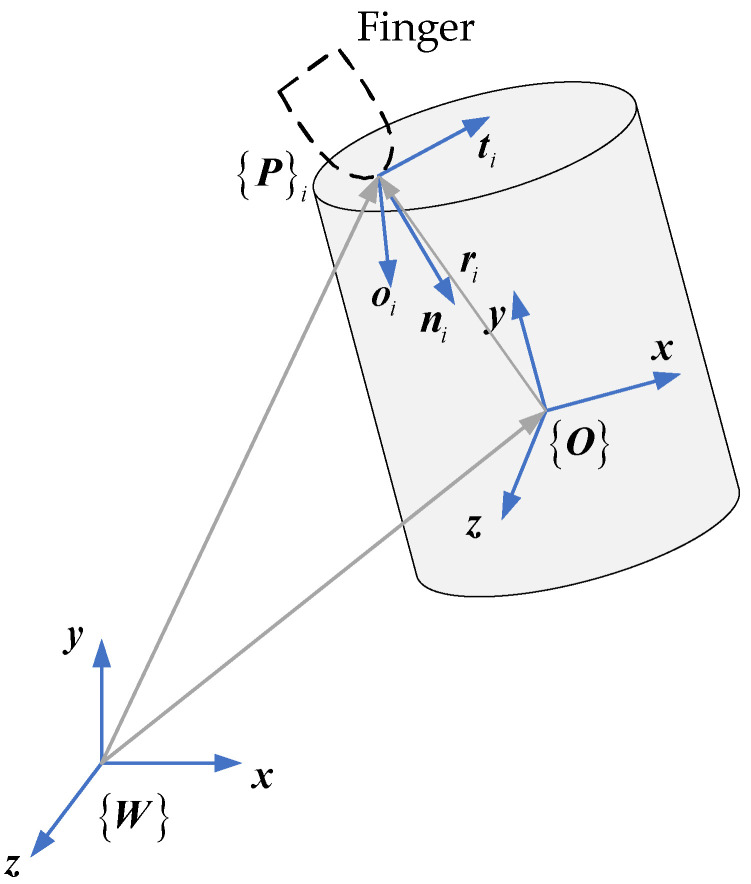
Point contact friction model.

When a finger contacts an object, the centroid position is the origin of reference coordinates. The contact between the finger and the object is regarded as a mapping between the force exerted by the finger on the contact point and the torque formed around the center of mass [28]. Object coordinate systems are based on the centroid and a set of coordinate systems based on each contact position, as shown in Figure 1.

For any contact point P, let the fingertips of the multi-fingered hand be locally prominent and incompressible. The contact point is fixed in place on the object. The contact between fingers and the object is frictional point contact. The contact force is $f_{i}$, and the direction of the normal force component is $n_{i}$. The following requirements must be met by the force applied to a body.

(1) $$\|f_{i}-\left(f_{i}\cdot n_{i}\right)n_{i}\|\leq \mu \left(f_{i}\cdot n_{i}\right)$$

For the frictional point contact model, the wrench of each point is a combination of the force and its moment about the origin of the object coordinates, and the wrench can be expressed as

(2) $$w_{i}=\left(\begin{array}{c} f_{i}\\ \tau_{i} \end{array}\right)=\left(\begin{array}{c} f_{i}\\ r_{i}\times f_{i} \end{array}\right)$$

where $r_{i}$ represents the position vector of the i contact point in the object coordinates relative to the centroid. The grasp wrench is three-dimensional for two-dimensional objects, whereas, for three-dimensional objects, the grasp wrench is six-dimensional.

Assume there is no relative slip at the contact points. By applying appropriate frictional forces in different directions, the multi-fingered hand can meet the requirements of either not moving or resisting arbitrary external forces. A grasp that satisfies this condition is termed force closure, meaning that any external wrench can be equilibrated by a non-negative combination of contact wrenches [29].

### 2.2. Grasp Analysis

Salisbury [30] has proven that a necessary and sufficient condition for achieving force closure in multi-fingered grasps is the set of basic wrenches from all contact points positively spanning the entire space. This means that given a set of n primitive contact wrenches, a necessary and sufficient condition for force closure is that a strictly positive linear combination of the primitive wrenches is zero, and the primitive wrenches span the whole wrench space [23].

Force closure can be calculated directly by the above condition, but it is quite complicated. Therefore, this section proposes a simplified method based on the actual configuration of multi-fingered dexterous hands. The accurate analysis and verification of force closure are essential for ensuring the operational and resisting capabilities of the multi-fingered hand, which is a primary goal in grasp planning. It is evident from the configuration and grasp analysis of human hands that the thumb, with its greater flexibility and supporting role, is crucial for further object manipulation. In anthropomorphic multi-fingered hands, similar to human hands, the thumb is positioned separately from the other fingers. Thus, all grasp configurations must involve the thumb, which is critical to ensuring stability during grasping, and its higher degree of freedom compared to the other fingers aids in performing additional tasks. Cutkosky categorized precise grasps into two cases: elongated grasps with two virtual fingers and radial symmetric grasps with three, where virtual fingers act similarly to single fingers [31].

Therefore, we consider the thumb and other fingers occupying two sides of the contact space, defining relative contact planes. For two-fingered frictional contact, force closure can only be achieved in a plane, and force closure is satisfied when the line connecting the two contact points is within both friction cones, a scenario also referred to as antipodal grasping. This can be extended to multi-fingered grasps in space, where the thumb is on one side and the friction cone of the thumb intersects with the triangle formed by the contact points of the other fingers, which cover most cases of precise grasping. Our algorithm is based on this grasp mode.

Therefore, we divide the thumb and other fingers into two sides of the contact space, establishing a relative contact plane. Force-closure conditions can only be met in a plane for a two-finger grasp with friction contact. If the line connecting the two contact points is within the friction cones, the force-closure condition is satisfied, and these types of grasp are antipodal grasps [22]. Extending this to multi-finger grasps in space, we define an antipodal grasp as a scenario where, with the relative contact planes present and the thumb on one side, the friction cone of the thumb intersects with a triangle that the other contact points create. This situation encompasses the most precise grasping scenarios for humanoid robot hands.

It has been demonstrated that in any n-dimensional Euclidean space $E^{n}$, at least n+1 vectors are required to achieve positive spanning [32]. Therefore, for force closure in three-dimensional space with frictional point contact, four fingers are sufficient. However, three fingers can also achieve force closure in three-dimensional space, representing a particular case for objects with specific symmetrical shapes, such as spheres and cubes. Geometric calculation algorithms for three-finger force-closure grasps have been developed, proving the necessary and sufficient conditions for force closure in rigid bodies constrained by three contact points [16]. However, the four-finger force-closure algorithm is mainly based on nonlinear programming.

This section analyzes four-fingered grasps and studies force-closure conditions in three-dimensional space. The calculations are significantly more complex since planar force-closure grasping involves a two-dimensional wrench space, and three-dimensional force closure involves a six-dimensional wrench space. Therefore, to simplify the calculations, we transform the force-closure conditions in the spatial domain to planar force-closure conditions. Meanwhile, achieving a non-marginal equilibrium is sufficient for the four-finger force closure. When the four-finger force equilibrium is achieved, the force closure is necessary for any more significant coefficients of friction. Based on this, we transform the force-closure condition to the force-equilibrium condition.

### 2.3. A Sufficient Condition for Four-Finger Force-Closure Grasps of 3-D Objects

Assume that in a multi-fingered hand model with four fingers, the contact points are denoted as $P_{1},\;P_{2},\;P_{3},\;P_{4}$, where $P_{1}$ represents the thumb contact point and $P_{2},\;P_{3},\;P_{4}$ represent the contact points of the other fingers. The relative positions of these contact points can be categorized into two cases—coplanar and non-coplanar.

When contact points are located inside the same plane, the contact force can only generate moments within that plane and cannot counteract external moments within that plane. This cannot achieve force closure in the grasp.

When contact points are non-coplanar, the lines between the points can form a tetrahedron.

The planes of the tetrahedron can be called the contact planes, and $P_{1}$ is the vertex that produces a total of three contact planes, which are expressed as $P_{123},\;P_{124},\;P_{134}$.

When the contact force components within the plane $P_{123},\;P_{124},\;P_{134}$ satisfy force closure, taking plane $P_{123}$ as an example, assume that any force wrench within the plane is denoted by $w_{1}=\left[F_{1}\;M_{1}\right]$. The projection of the contact force at points $P_{1},\;P_{2},\;P_{3}$ onto the plane is $f_{S1},\;f_{S2},\;f_{S3}$. The moments generated about the coordinate origin are $M_{S1},\;M_{S2},\;M_{S3}$. Then, there must exist

(3) $$\begin{array}{c} f_{S1}+f_{S2}+f_{S3}+F_{1}=0\\ M_{S1}+M_{S2}+M_{S3}+M_{1}=0 \end{array}$$

In the same way, there must also be contact forces in planes $P_{124}$ and $P_{134}$ that can quilibrate any wrench within that plane.

Three lines intersecting at point $P_{1}$ are not coplanar in space, forming a non-orthogonal system. The following proof demonstrates that the wrench within these three contact planes can represent any wrench in space.

Firstly, we define a four-dimensional linear space. For a basis in this space, we can choose four points: $p_{1},\;p_{2},\;p_{3},\;p_{4}$. These four points can be written as a linear combination to represent any point in space.

(4) $$\begin{array}{c} P_{a}=a_{1}p_{1}+a_{1}p_{2}+a_{1}p_{3}+a_{1}p_{4}\\ P_{b}=b_{1}p_{1}+b_{1}p_{2}+b_{1}p_{3}+b_{1}p_{4} \end{array}$$

where $a_{i}$ and $b_{i}$ are scalars, and the sum of $a_{i}$ and $b_{i}$ is 1. Next, we can represent the lines in the Grassmann space using the exterior product of the points. They are also the six sides of the tetrahedron.

(5) $$\begin{array}{c} L_{1}=p_{1}\times p_{2}\;L_{2}=p_{1}\times p_{3}\;L_{3}=p_{1}\times p_{4}\\ L_{4}=p_{2}\times p_{3}\;L_{5}=p_{2}\times p_{4}\;L_{6}=p_{3}\times p_{4} \end{array}$$

Therefore, six mutually independent lines can be constructed starting from these four points. These six edge lines can be combined by linear combinations to represent any line. Any line L can be represented by expanding and simplifying the cross product of two points on the chosen line [33].

(6) $$\begin{array}{c} P_{a}=a_{1}p_{1}+a_{1}p_{2}+a_{1}p_{3}+a_{1}p_{4}\\ P_{b}=b_{1}p_{1}+b_{1}p_{2}+b_{1}p_{3}+b_{1}p_{4} \end{array}$$

These six lines can serve as the basis for a 6D wrench space. The contact wrenches are a representation of applying force $f_{i}$ at a point on line L. Therefore, for any external wrench $W={\left[F\;M\right]}^{T}$ in this space, there must be forces that satisfy $w_{1}+w_{2}+w_{3}=W$. In other words, the three lines intersecting $P_{1}$ construct the basis for a 6D wrench space.

As a result, when components of the grasping forces are in a state of force closure within these three contact planes, the wrench basis within these three planes can represent any wrench in the space. At the moment, the four points must achieve spatial force-closure grasps. In other words, given a rigid body in space subjected to constraints with friction at four contact points, a sufficient condition for force closure is that the four points are non-coplanar and the contact forces are in a state of force closure within three contact planes.

## 3. Algorithm

### 3.1. Contact Force Analysis in Contact Plane

For a system with n wrenches $w_{1},\cdots ,w_{n}$, if the system origin is located inside the convex hull formed by all force screw axes, the system constitutes force-closed grasping [34]. Force equilibrium, however, is a weaker constraint compared to force closure. If the origin of the system is inside the convex hull formed by the force screw axes, the system achieves force equilibrium [23]. This implies that if the system origin is on the surface of the convex hull formed by wrenches, it is still considered force equilibrium grasping. Thus, there is a containment relationship between force closure and force equilibrium. A grasp satisfying the force-closure condition will always fulfill the force-equilibrium condition, but a force equilibrium system is not necessarily force-closed. Force closure is achieved if the grasp can form a non-critical force equilibrium.

**Figure 2:**
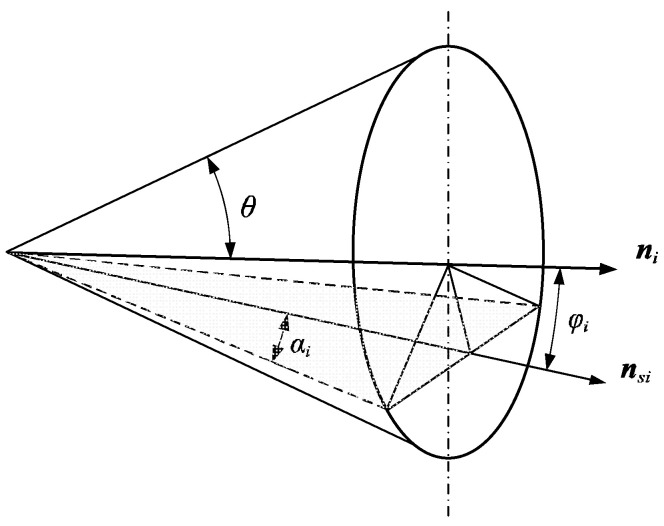
Friction cone and projection.

Force-closure grasping belongs to the non-critical state of force equilibrium grasping [35]. For grasping that satisfies force equilibrium, force closure can be achieved simply by increasing the friction coefficient at the contact points. Therefore, for grasping that has achieved force equilibrium, if the coefficient of friction increases, a non-critical force equilibrium can be achieved, that is, force closure. To reduce the computational load, we can first consider transforming the force-closure conditions for a four-finger grasp in space into two-dimensional force-closure conditions, and then simplify the two-dimensional force closure to force-equilibrium conditions. There are three possible relationships between the friction cone between the contact planes and the contact points: intersecting, tangent, and disjoint. When a friction cone intersects with a contact plane, the intersecting portion is called the friction fan [36], as shown in Figure 2.

Assume the normal vectors projected within the contact plane are $n_{1},\;n_{2},\;n_{3}$. Define the normal vector of the contact plane as

(7) $$n_{k}=\frac{\left(n_{2}-n_{1}\right)\times \left(n_{3}-n_{2}\right)}{\left|n_{2}-n_{1}\right|\left|n_{3}-n_{2}\right|}$$

The angle between the $n_{1}$ and the vector $n_{k}$ is

(8) $$\lambda_{i}=\arccos\left(n_{k}\cdot n_{i}\right)$$

To ensure that a force acts on the contact point within the contact plane, the friction cone must intersect with the contact plane. Therefore, the condition that follows must be met for the angle between the normal vector and the contact plane.

(9) $$\left|\frac{\pi }{2}-\lambda_{i}\right|=\phi_{i}<\arctan\;\mu =\theta$$

The frictional angle within the plane is

(10) $$\alpha_{i}=\arcsin\sqrt{\sin^{2}\theta -\cos^{2}\theta \tan^{2}\phi_{i}}$$

After projecting the normal vector onto the contact plane, it can obtain

(11) $$n_{si}=\frac{n_{i}-n_{ki}\cos\lambda_{i}}{\sin\lambda_{i}}$$

At this point, the boundary vectors of the friction fan are

(12) $$\begin{array}{c} n_{i1}=\frac{n_{si}\cos\alpha_{i}+\left(n_{ki}\times n_{si}\right)\sin\alpha_{i}}{\left|n_{si}\cos\alpha_{i}+\left(n_{ki}\times n_{si}\right)\sin\alpha_{i}\right|}\\ n_{i2}=\frac{n_{si}\cos\alpha_{i}-\left(n_{ki}\times n_{si}\right)\sin\alpha_{i}}{\left|n_{si}\cos\alpha_{i}-\left(n_{ki}\times n_{si}\right)\sin\alpha_{i}\right|} \end{array}$$

A sufficient condition for force closure is non-marginal equilibrium. Thus, grasps achieve equilibrium with non-zero forces for some friction coefficients and achieve force closure for any more strictly significant friction coefficient [24].

The necessary and sufficient condition for force equilibrium grasping with three contact points is that three non-zero contact forces exist within the friction cone that spans the entire plane, and the lines of action of these forces intersect at a single point [32]. However, due to high computational complexity, this is rarely used directly for calculating three-finger equilibrium grasping.

According to the properties of vector linear algebra, any two non-collinear vectors in a two-dimensional plane can form a basis for that plane. Thus, two cases exist for force equilibrium in the contact plane: two-point force equilibrium and three-point force equilibrium.

### 3.2. Force Equilibrium Grasps

The necessary and sufficient condition for force equilibrium for a two-point contact grasp with friction is that the line connecting the two contact points lies within both friction cones [16]. Let two contact points be $P_{1}$ and $P_{2}$, and the friction fan boundaries within the contact plane be $n_{11},\;n_{12},\;n_{21},\;n_{22}$. The following constraint can be used to represent the condition.

(13) $$\left\{\begin{array}{c} n_{11}\times P_{1}P_{2}>0\\ n_{12}\times P_{1}P_{2}<0\\ n_{21}\times P_{2}P_{1}>0\\ n_{21}\times P_{2}P_{1}<0 \end{array}\right.$$

Let $f_{si},\;f_{sj},\;f_{sk}$ be the projection of the contact force at points $P_{i},\;P_{j},\;P_{k}$ within the contact plane, respectively. Since the plane must be located inside the object, the plane forces should cancel each other, and the forces in each plane should take the moment to $C_{k}$, then it must have

(14) $$f_{si}\times P_{k}P_{i}+f_{sj}\times P_{k}P_{j}=0$$

When $P_{k}P_{i}\times f_{si}=P_{k}P_{j}\times f_{sj}=0$, $f_{si}$ and $f_{sj}$ both pass through contact point $P_{k}$. Since $P_{k}$ is the vertex of the friction cone, equilibrium cannot be valid Therefore, the moments of the internal forces about any contact point must be in opposite directions.

(15) $$Sgn\left(f_{si}\times P_{k}P_{i}\right)\cdot Sgn\left(f_{sj}\times P_{k}P_{j}\right)<0$$

Let the directions of three forces $f_{si},\;f_{sj},\;f_{sk}$ be $n_{si},\;n_{sj},\;n_{sk}$, respectively. Then, we have

(16) $$Sgn\left(n_{si}\times P_{k}P_{i}\right)\cdot Sgn\left(n_{sj}\times P_{k}P_{j}\right)<0$$

Since the projections of the contact forces at each point onto the plane lie within the corresponding friction cone, at least one moment of the boundary vectors $n_{i1}$ and $n_{i2}$, with respect to point $P_{k}$, should have the same direction as the moment of vector $n_{i}$ about $P_{k}$. That is,

(17) $$Sgn\left(n_{i1}\times P_{k}P_{i}\right)\cdot Sgn\left(n_{si}\times P_{k}P\right)>0$$

(18) $$Sgn\left(n_{i2}\times P_{k}P_{i}\right)\cdot Sgn\left(n_{si}\times P_{k}P_{i}\right)>0$$

At least one of Equations (17) and (18) should be true. Therefore, at least one of the following two formulas should be true.

(19) $$Sgn\left(n_{i1}\times P_{k}P_{i}\right)\cdot Sgn\left(n_{sj}\times P_{k}P_{j}\right)<0$$

(20) $$Sgn\left(n_{i2}\times P_{k}P_{i}\right)\cdot Sgn\left(n_{sj}\times P_{k}P_{j}\right)<0$$

Similarly, at least one of the moments of $n_{j1}$ and $n_{j2}$ with respect to point $P_{k}$ should have the same direction as the moment of $n_{i}$ about $P_{k}$. Therefore, to achieve force equilibrium in the plane, at least one of the following inequalities must be satisfied.

(21) $$Sgn\left(n_{i1}\times P_{k}P_{i}\right)\cdot Sgn\left(n_{j1}\times P_{k}P_{j}\right)<0$$

(22) $$Sgn\left(n_{i1}\times P_{k}P_{i}\right)\cdot Sgn\left(n_{j2}\times P_{k}P_{j}\right)<0$$

(23) $$Sgn\left(n_{i2}\times P_{k}P_{i}\right)\cdot Sgn\left(n_{j1}\times P_{k}P_{j}\right)<0$$

(24) $$Sgn\left(n_{i2}\times P_{k}P_{i}\right)\cdot Sgn\left(n_{j2}\times P_{k}P_{j}\right)<0$$

where i, j, and k in the inequality cycle through 1, 2, and 3.

To further reduce the amount of computation, we consider simplifying the force-equilibrium condition by using the normal force in the contact plane. Let $n_{i},\;n_{j},\;n_{k}$ represent the normal force directions at points $P_{i},\;P_{j},\;P_{k}$ within the plane. We consider the friction cone to be approximately perpendicular to the bottom surface of the tetrahedron. Then, a sufficient condition for the three points to satisfy the force equilibrium is as follows:

(1) The normal vectors satisfy the following relationship:

(25) $$\left\{\begin{array}{l} Sgn\left(n_{j}\times n_{i}\right)\cdot Sgn\left(n_{j}\times n_{k}\right)<0\\ Sgn\left(n_{i}\times n_{j}\right)\cdot Sgn\left(n_{i}\times n_{k}\right)<0 \end{array}\right.$$

(2) The vectors formed by the normal vectors and the contact points satisfy the following relationship:

(26) $$\left\{\begin{array}{l} Sgn\left(n_{i}\times P_{k}P_{i}\right)\cdot Sgn\left(n_{j}\times P_{k}P_{j}\right)<0\\ Sgn\left(n_{i}\times P_{j}P_{i}\right)\cdot Sgn\left(n_{k}\times P_{j}P_{k}\right)<0\\ Sgn\left(n_{j}\times P_{i}P_{j}\right)\cdot Sgn\left(n_{k}\times P_{i}P_{k}\right)<0 \end{array}\right.$$

When condition (1) is satisfied, it indicates that the torque generated by $n_{1}\times n_{2}$ on the object is opposite to that of $n_{1}\times n_{3}$, and the torque generated by $n_{2}\times n_{1}$ on the object is opposite to $n_{2}\times n_{3}$.

If there is $n_{1}\times n_{2}=-k\cdot \left(n_{1}\times n_{3}\right),k\geq 0$, we have

$$n_{1}\times n_{2}+k\cdot \left(n_{1}\times n_{3}\right)=n_{1}\times \left(n_{2}+k\cdot n_{3}\right)=0$$

If there is $n_{2}+k\cdot n_{3}=0$, then $n_{2},\;n_{3}$ are in opposite directions. Considering the presence of the friction cone, $n_{2},\;n_{3}$ span the entire plane.

If there is $n_{2}+k\cdot n_{3}\neq 0$, then the moments of force $n_{1}$ and $n_{2}+k\cdot n_{3}$ are parallel.

Let us prove by contradiction that $n_{1}$ is opposite to $n_{2}+k\cdot n_{3}$.

$n_{1}$ and $n_{2}+k\cdot n_{3}$ are in the same direction. Let $n_{1}=s\cdot \left(n_{2}+k\cdot n_{3}\right)$, where s>0, we have $n_{1}\times n_{2}=s\cdot \left(n_{2}+k\cdot n_{3}\right)\times n_{2}=s\cdot k\cdot n_{3}\times n_{2}$.

Since s⋅k≥0, the above statement contradicts the known condition that $n_{2}\times n_{1}$ and $n_{2}\times n_{3}$ are in opposite directions. Therefore, the assumption is incorrect, and thus $n_{1}$ and $n_{2}+k\cdot n_{3}$ are in opposite directions.

Let $n_{2}+k\cdot n_{3}=-s\cdot n_{1},s\geq 0$.

Since $s\cdot n_{1}+n_{2}+k\cdot n_{3}=0,s,\;k\geq 0$, $n_{1},\;n_{2},\;n_{3}$ span the entire plane positively.

When condition (2) is satisfied, let the normal contact forces at the three points in the plane be $f_{i}=an_{i}$, $f_{j}=bn_{j}$, and $f_{k}=cn_{k}$.

For any point O on the plane, the moments of the three forces relative to point O are

$$M_{O}=f_{n1}\times OC_{1}+f_{n2}\times OC_{2}+f_{n3}\times OC_{1}=f_{n1}\times C_{2}C_{1}+f_{n3}\times C_{2}C_{3}+\left(f_{n1}+f_{n2}+f_{n3}\right)\times OC_{2}$$

From condition (2), we know that $f_{n1}\times C_{2}C_{1}$ is opposite to $f_{n3}\times C_{2}C_{3}$, $f_{n1}\times C_{3}C_{1}$ is opposite to $f_{n2}\times C_{3}C_{2}$, and the module of $C_{2}C_{3}$ is $C_{23}$, while the module of $C_{3}C_{1}$ is $C_{31}$, and the module of $C_{2}C_{1}$ is $C_{21}$. If $a=C_{23}$, $c=C_{31}$, $c=C_{21}$, then there is

$$\begin{array}{c} f_{n1}\times C_{2}C_{1}=-f_{n3}\times C_{2}C_{3}\\ f_{n1}\times C_{3}C_{1}=-f_{n2}\times C_{3}C_{2}\\ f_{n3}\times C_{1}C_{3}=-f_{n2}\times C_{1}C_{2} \end{array}$$

Thus, there are

$$\left(f_{n1}+f_{n3}\right)\times C_{2}C_{1}=-f_{n3}\times C_{2}C_{3}+f_{n3}\times C_{2}C_{1}=f_{n3}\times C_{3}C_{1}$$

$$\left(f_{n1}+f_{n2}+f_{n3}\right)\times C_{2}C_{1}=f_{n3}\times C_{3}C_{1}+f_{n2}\times C_{2}C_{1}=0$$

Similarly, there are

$$\left(f_{n1}+f_{n2}+f_{n3}\right)\times C_{3}C_{1}=0$$

$$\left(f_{n1}+f_{n2}+f_{n3}\right)\times C_{3}C_{2}=0$$

Since $P_{1},P_{2},P_{3}$ are not collinear, there are $\left(f_{n1}+f_{n2}+f_{n3}\right)=0$

It can be seen that for any point O on the plane, there is a contact force at the three contact points so that their combined force and moment at point O are simultaneously zero, so the grasp is force equilibrium. Some grasps do not satisfy the above conditions but are force equilibrium, which is the cost of simplifying the algorithm.

**Figure 3:**
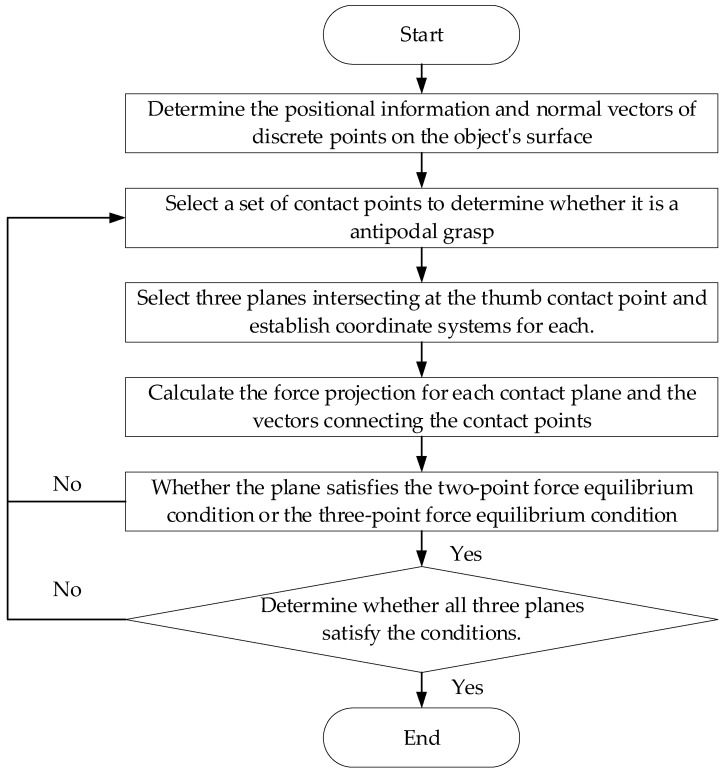
The flow chart of the force-closure algorithm.

A specific flow chart of the complete force-closure algorithm is shown in Figure 3.

## 4. Application Examples

**Figure 4:**
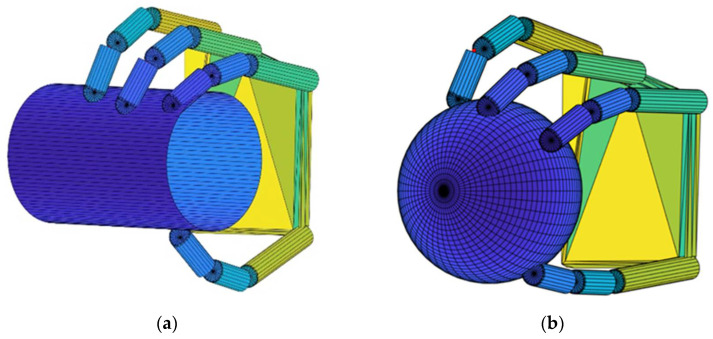
Four-finger grasping models. (**a**) Cylinder grasp. (**b**) Ball grasp.

To verify the efficiency of the recommended spatial four-finger force-closure grasping algorithm, force-closure calculations were performed for spheres and cylinders, as shown in Figure 4.

First, the object surface was discretized to determine positions of the contact point and directions of the normal force. The contact points’ coordinates are identified by position vectors relative to a coordinate system centered on the object centroid, with normal forces always pointing inward. We obtain an initial set of points by intersecting the workspace of the fingers with the three-dimensional model of the object. This ensures that all points in the search area to be optimized before optimization are on the object’s surface and are reachable by the fingers.

For a cylinder with a diameter of 60 cm, a height of 100 cm, and a friction coefficient of 0.6, after discretizing the surface and randomly selecting 10,000 contact point sets, 302 force-closure grasps were obtained, with the computation time being 103 milliseconds. For a sphere with a radius of 50 cm and a friction coefficient of 0.4, the first contact point was fixed, and the three remaining contact points were randomly chosen in the opposite grasp plane, resulting in 10,000 contact point sets. This yielded 1178 force-closure grasps, with the algorithm computing time being 283 milliseconds. The calculation results are shown in Table 1.

The force-closure algorithm entirely employs vector-based algebraic computations. It avoids high-order matrix decompositions and iterative calculations, thus reducing computation time and improving efficiency. Calculations for several typical geometric shapes show that it can find most solutions that satisfy the force-closure condition, confirming the algorithm’s effectiveness. This algorithm effectively analyzes and assesses spatial force-closure problems in four-finger grasping, facilitating online planning and real-time applications for grasping.

## 5. Discussion

The force-closure algorithm in this paper is primarily based on geometric principles, with conditions being checked using vector relationships. It involves multiple checks to directly exclude points that do not satisfy the force-closure conditions, resulting in low complexity. The necessary and sufficient condition for force closure is that the feasible external force wrenches completely fill the wrench space. For computational convenience, this is often expressed as the origin being inside the convex hull of all possible force wrenches. Most force-closure algorithms are based on this principle. In practice, the nonlinear friction cone is first linearized into forms like hexagonal or octagonal pyramids. For example, linearizing the friction cone at contact points into a hexagonal pyramid requires constructing a convex hull from 24 six-dimensional wrenches, which is complex in high-dimensional space. Additionally, it is necessary to determine whether the coordinate origin is inside the convex hull, typically solved through linear programming problems. Given the high number of force wrenches after friction cone linearization, the convex hull may have many faces, significantly increasing the computational burden. Therefore, each set of grasping points requires repeating the complete convex hull construction and linear programming process.

The improved ray-shooting method in the literature [19] is used to test the calculation results obtained by this algorithm, and the friction cone is linearized into a 10-sided polyhedral cone. The results prove that the contact point set obtained by this algorithm can achieve force-closure grasp. The algorithm in this paper overlooks a portion of feasible solutions due to the constraints and approximations in the grasping conditions. However, this algorithm can quickly filter out the infeasible points through simple discrimination and obtain the majority of feasible solutions. In the future, we also need to optimize the stability and operability of the grasping based on the configuration of the multi-fingered dexterous hand.

## 6. Conclusions

A novel method to calculate the force-closure grasps of three-dimensional objects with multi-fingered hands is proposed. The approach transforms the three-dimensional force-closure condition into a planar condition, requiring only vector operations. This method is less sophisticated, making it appropriate for grasping in real time. Finally, force-closure computations verify the efficiency of the suggested approach for several common geometric forms. The results of multiple experiments indicate that the proposed algorithm can obtain most force-closed grabs within a few hundred milliseconds. In the future, we will further optimize the contact points based on this force-closure algorithm, incorporating grasp quality metrics to identify contact points that meet the specified task requirements. Additionally, we will integrate the pose of the wrist and multi-fingered hand into the grasp planning.

## Figures and Tables

**Table 1:** Calculation results.

Object	Antipodal Grasps	Force-Closure Grasps	Force-Closure Grasp Ratio	Computation Time (ms)
Cylinder	842	302	35.87%	103
Ball	2455	1178	47.98%	283

## Data Availability

There are no data to be shared.
